# How to manage herpes zoster ophthalmicus

**Published:** 2020-03-30

**Authors:** Stephen Tuft

**Affiliations:** 1 Consultant Ophthalmologist Moorfields Eye Hospital, London, UK.

**Herpez zoster ophthalmicus is a severe variant of shingles (herpes zoster), which occurs when the immune system is weakened and the virus responsible for chickenpox reactivates.**

**Figure 1:**
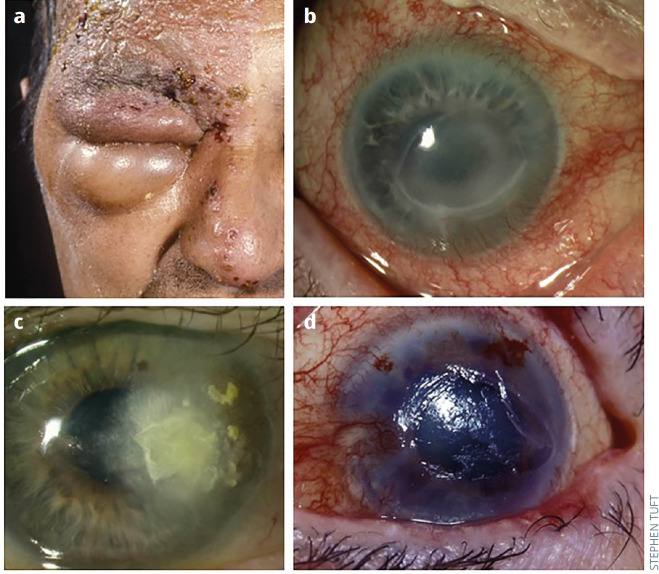
Acute herpes zoster ophthalmicus (HZO) with blisters and eyelid swelling (a). Chronic corneal epithelial defect in an anaesthetic cornea (b). Corneal anaesthesia complicated by secondary corneal calcification (c) and vascularisation (d).

Herpes zoster (shingles) is an infection caused by re-activation of the varicella zoster virus, which causes chickenpox. Primary infection usually happens in childhood. After the primary infection, the patient is immune to the virus, which then lies inactive in the dorsal root ganglia or cranial ganglia. Eventually, as the patient's natural immunity reduces as a result of ageing or disease, the virus can reactivate and spread to the skin, via the afferent nerves, to cause herpes zoster. By the time they are 40 years of age, the vast majority (99.6%) of individuals worldwide have antibodies to varicella zoster virus and are therefore at risk of herpes zoster.^1^

About one-third of individuals will get herpes zoster in their lifetime. Herpes zoster can appear at any age, although there is a strong tendency for it to develop after 60 years of age due to natural weakening of the immune system. There has been a worldwide increase in the prevalence of herpes zoster. The reason for this is not known; however, with an ageing population, the incidence is likely to increase.

**“About one-third of individuals will get herpes zoster in their lifetime.”**

The majority of individuals who get herpes zoster are otherwise healthy. However, it is more common in immunocompromised individuals (e.g., after chemotherapy, immunotherapy or oral steroids) or with some diseases (e.g., diabetes mellitus or cancer). In some regions, herpes zoster may signal underlying immunosuppression from HIV/AIDS, especially in young adults; these patients are also at risk of severe complications and systemic involvement. Recurrent attacks of herpes zoster are uncommon, but they are a feature of HIV/AIDS.

## Clinical presentation

Herpes zoster most frequently involves the chest, abdomen, face or genitals. The distribution corresponds to the distribution of the nerve (dermatome) that has been affected. The disease does not cross the midline, but two adjacent dermatomes on the same side can be involved. Herpes zoster ophthalmicus (HZO) is a particularly severe variant of herpes zoster and accounts for about 20% of all cases. The eye is not always involved, but there is a particularly high risk (50%) of ocular involvement if the first division of the fifth cranial (trigeminal) nerve is affected, with vesicles extending to the tip of the nose, known as Hutchinson's sign (Figure 1).^1^,^2^

The clinical signs of HZO are usually preceded by tingling over the affected area, and patients generally feel unwell and have a headache. After 2–3 days, a painful rash develops, consisting of blisters that ooze fluid for 3–5 days. The blisters then dry out and form scabs that heal after four weeks. The skin remains painful until the rash has gone.

Post-herpetic neuralgia sometimes develops. It involves pain, itching, and burning over the area of the initial rash that is still present after three months. This develops in about 13% of all cases, and half of individuals over 70 years will still have pain from it after a year. The incidence of post-herpetic neuralgia increases with age of onset of HZO, with eye involvement, and with the severity of the initial attack. It can be debilitating, preventing sleep and daily activities, and severely affect quality of life.

HZO can affect all parts of the eye, with an onset of disease 2–4 weeks after the first appearance of the rash. Anterior segment complications can be severe. They include dendritic keratitis, secondary corneal anaesthesia, persistent epithelial defect, secondary infection, stromal neovascularisation, and corneal opacity (Figure 1).

Patients with HIV/AIDS, in particular, can develop disfiguring scarring and pigmentation of the face and lids. There may also be chronic granulomatous uveitis, iris atrophy, scleritis, and acute retinal necrosis. Vasculitis is less common but can cause ischaemic optic neuropathy, extraocular muscle palsies and a contralateral stroke.

Varicella zoster virus is the most common cause of acute retinal necrosis. However, acute retinal necrosis is not restricted to patients who have had HZO, which suggests that blood, rather than nerves, are responsible for the spread of the virus to the eye.

## Diagnosis

The appearance of HZO is very characteristic, and this gives the diagnosis in almost all cases. However, tingling or pain exacerbated by light touch may develop several days before the rash or, rarely, a rash may never develop (zoster sine herpete). In these patients, identifying the cause of the neuralgia can be difficult. Herpes virus DNA can sometimes be detected in the tear film by polymerase chain reaction (PCR), although this is an expensive test and not widely available. As the majority of adults have had chickenpox, they will have antibodies against varicella zoster virus and serology is rarely helpful. It is not necessary to perform extensive investigation for underlying diseases, unless HIV/AIDS is suspected.

## Differential diagnosis

Herpes simplex infection of the lid and cornea can mimic HZO, especially if there is bacterial superinfection of severe atopic dermatitis (eczema herpeticum). Contact dermatitis from plants, or a reaction to locally applied medications, can mimic HZO. Consider herpes simplex if patients have had multiple recurrences of ‘shingles’. A bilateral rash is unlikely to be HZO.

## Treatment

Medical staff should wear gloves if touching skin with blisters; however, if they have had chickenpox in the past, they are not at risk of infection. Wash the affected skin with soap and water and pat dry, cover with a loose dressing, and use a dry cold compress if available. Don't use dressings that can stick to the skin. Antibiotics are not necessary unless secondary skin infection is suspected, and don't use topical antivirals on the skin.

There is no agreed consensus for the medical management of HZO. If a patient is seen within 72 hours of the onset of the blisters, they should be given a course of oral aciclovir tablets: 800 mg five times daily for seven days (alternatives are either valaciclovir 1 g or famciclovir 500 mg, three times a day). Provide this treatment at the start of any new ocular involvement. Intravenous aciclovir should be considered for individuals with HIV/AIDS to reduce the risks of disseminating varicella zoster infection. Antiviral treatment reduces the risk of chronic ocular complications by 20% to 30%. It does not prevent post-herpectic neuralgia but reduces the duration of the pain by about 50%.

Dendritic keratitis and uveitis may indicate chronic HZV activity. Keratitis should be treated with topical aciclovir 3% ointment or ganciclovir 0.15%, five times daily for at least 5 days, reducing to twice daily until the dendrites have resolved. Add a topical steroid if there is stromal disease or uveitis. Slow tapering of topical steroid may be required, and some individuals need long-term drops once a day to control inflammation. Long-term prophylactic antiviral treatment (oral aciclovir 400 mg twice a day) is often used to treat these complications and prevent reactivation of the varicella zoster virus in the eye, but this is not yet evidence based.

Patients can develop complete corneal anaesthesia after HZO, known as neurotrophic keratitis. This can lead to sight loss, as the anaesthetic cornea is at risk of developing an epithelial defect, secondary infection, vascularisation and scarring. Frequent lubricating drops are helpful to prevent epithelial breakdown. A chronic epithelial defect may heal with a temporary tarsorrhaphy, bandage contact lens or amniotic membrane graft. Unfortunately, a proportion of patients eventually require a permanent tarsorrhaphy or conjunctival flap to protect the eye. Severe post-herpetic neuralgia may be helped by giving oral analgesics (paracetamol, short-acting narcotics), anticonvulsants (e.g., gabapentin) or tricyclic anti-depressants. Acute retinal necrosis requires specialist management and patients should be referred to a tertiary centre if possible. Treatment includes intravenous aciclovir 10–15 mg/kg for 2 weeks, followed by oral valaciclovir 1 g three times daily for a further 6 weeks.

## Follow-up

Corneal anaesthesia is a common feature of HZO, and progressive corneal disease may pass unnoticed. Review patients periodically or ask them to monitor their vision themselves and seek help if it deteriorates. Patients receiving topical steroid treatment should be monitored for complications.

## Prevention and vaccination

A vaccine against chickenpox is available in many countries. This uses a live, attenuated virus, and is also used at a higher dose to prevent (not treat) herpes zoster. Because it is a live virus, do not give this vaccine to people who have impaired immunity (e.g., people with HIV/AIDS). A recombinant virus vaccine is also available to treat herpes zoster and, as it does not contain a live virus, it can be used in immunocompromised patients. Both vaccines boost cell-mediated immunity against varicella zoster virus, which then reduces the risk of reactivation. Regional guidelines vary, but vaccination against herpes zoster is usually only recommended for individuals over 60 years of age. Both vaccines are safe to use in patients who have had chickenpox and individuals do not need to be tested for immunity against the virus before they are vaccinated. A large randomised-controlled clinical trial found that, after three years, the live attenuated vaccine had reduced the incidence of herpes zoster amongst adults over 60 years of age by 51% and the burden of illness by 66.5%.^3^ A second observational study of the use of the same vaccine in patients over 60 years of age reported that it reduced the risk of eye involvement (HZO) by almost two thirds (hazard ratio 0.37).^4^

Unfortunately, the effect of vaccination only lasts for about eight years, and the vaccine is less effective in patients older than 70 years. The recombinant vaccine is more effective than the live attenuated vaccine, and it reduces the incidence of herpes zoster and post-herpetic neuralgia by 90%; it is now the preferred option in some countries.^5^ If these vaccines are available and recommended for use in your country, the current recommendations are to use the live attenuated vaccine to prevent chickenpox and the recombinant vaccine to prevent herpes zoster. Interestingly, vaccination in childhood does not seem to reduce the risk of herpes zoster in later life.

Cost is a consideration in the widespread introduction of vaccination against herpes zoster. Prophylactic low-dose oral aciclovir (400 mg twice daily) is a less effective alternative to vaccination for patients at high risk of herpes zoster.

## Patient counselling

A patient with HZO can spread chickenpox in non-immune individuals, but HZO cannot be transmitted directly to cause HZO in another person. Transmission of the virus typically requires direct contact with oozing skin. The risk of transmission stops when the rash crusts and stops leaking. Patients who are immunocompromised (e.g., those who have HIV/AIDS) are more contagious because they shed more virus particles. A person who has HZO blisters should avoid direct contact with babies less than 1 month of age, pregnant women who have not had chickenpox, and individuals with a suppressed immune system.
